# Robot-Assisted Mirror Rehabilitation for Post-Stroke Upper Limbs: A Personalized Control Strategy

**DOI:** 10.3390/s25185659

**Published:** 2025-09-11

**Authors:** Jiayue Chen, Zhongjiang Cheng, Yutong Cai, Shisheng Zhang, Chi Zhu, Yang Zhang

**Affiliations:** 1 Sino-German College of Intelligent Manufacturing, Shenzhen Technology University, Shenzhen 518118, China; 2310415038@stumail.sztu.edu.cn (J.C.); chengzhongjiang@sztu.edu.cn (Z.C.); 202200101094@stumail.sztu.edu.cn (Y.C.); zhuchi@sztu.edu.cn (C.Z.); 2 Shenzhen Institutes of Advanced Technology, Chinese Academy of Sciences, Shenzhen 518055, China; ss.zhang6@siat.ac.cn

**Keywords:** mirror rehabilitation therapy, upper-limb exoskeleton robot, attention mechanism, dynamic movement primitives, admittance control

## Abstract

To address the limitations of traditional mirror therapy in stroke rehabilitation, such as rigid movement mapping and insufficient personalization, this study proposes a robot-assisted mirror rehabilitation framework integrating multimodal biofeedback. By synchronously capturing kinematic features of the unaffected upper limb and surface electromyography (sEMG) signals from the affected limb, a dual-modal feature fusion network based on a cross-attention mechanism is developed. This network dynamically generates a time-varying mirror ratio coefficient λ, which is incorporated into the exoskeleton’s admittance control loop. Combining a trajectory generation algorithm based on dynamic movement primitives (DMPs) with a compliant control strategy incorporating dynamic constraints, the system achieves personalized rehabilitation trajectory planning and safe interaction. Experimental results demonstrate that, compared to traditional mirror therapy, the proposed system exhibits superior performance in bilateral trajectory covariance metrics, the mirror symmetry index, and muscle activation levels.

## 1. Introduction

Stroke is a debilitating neurological disorder that often leads to hemiplegia—paralysis or severe weakness on one side of the body—resulting in significant motor impairments. Compared to healthy individuals, stroke patients typically exhibit reduced grip strength, slower walking speed, and decreased knee joint strength [1]. Annually, approximately 2.41 billion people could benefit from rehabilitation during illness or injury [2]. Upper-limb rehabilitation is a critical domain in stroke recovery due to its profound impact on functional restoration and quality of life for individuals with neurological damage. Effective upper-limb rehabilitation significantly enhances motor function, muscle strength, and the capacity to perform activities of daily living (ADLs) [3].

While traditional unilateral ADL training provides therapeutic benefits for stroke patients, the motor skills acquired through unilateral training often fail to generalize effectively to bilateral limb function [4]. Bilateral training, however, activates the undamaged hemisphere to increase activation in the damaged hemisphere when both arms share a common movement intention, promoting neuroplasticity and motor control in the affected limb [5]. Therefore, bilateral training is essential for stroke rehabilitation.

Mirror therapy (MT) is a relatively novel stroke intervention focusing on movement of the unaffected limb while receiving mirrored visual feedback of that movement [6]. It primarily leverages motor imagery, where the brain’s motor cortex activates to some extent when imagining limb movement [7]. However, traditional MT relies predominantly on movement of the healthy limb and often neglects the residual motor capacity of the affected arm. This results in several limitations: it depends on symmetric movement reflexes and is largely ineffective for patients with severe impairments who are unable to move the affected limb [8,9]. Furthermore, since the affected limb does not actually move, the effectiveness in restoring real motor function may be reduced [10].

Robot-assisted mirror therapy represents an advanced modality of rehabilitation, particularly suitable for hemiplegic patients [11,12,13]. Bilateral robotic systems in this category facilitate training through customized trajectories involving coordinated movements of both the affected and unaffected limbs. For instance, the robot-assisted BFIAMT (Bilateral Force-Induced Arm Movement Trainer) [14] enables users to manipulate two conical handles symmetrically and smoothly via push–pull forces applied by both hands. This bilateral approach is compatible with mirror therapy, potentially enhancing recovery. The Mirror Movement Enabler (MIME) [15] employs a “leader–follower” mode, continuously guiding the paretic limb to mirror the position of the contralateral limb, with the movement speed and range controlled by the patient. Similarly, the EXO-UL7 [16] adopts a leader–follower strategy but provides only partial assistance, meaning that the affected limb is not forced into an exact mirror position. With robotic assistance, patients engaged in therapeutic games in a virtual environment and demonstrated significant post-treatment improvement [17,18]. These findings suggest that bilateral robots with leader–follower modes enable more effective training movements.

While existing robotic training paradigms help patients utilize residual motor capacity, they often lack contextually relevant, task-based assistance mimicking real-life ADLs, such as picking up a cup or opening a drawer. Engagement in ADL-related tasks has been shown to improve functional outcomes, foster independence, and boost rehabilitation confidence in stroke patients [19,20]. Wang et al. proposed a trajectory capture and tracking scheme leveraging Kinect sensors for upper-limb robot-assisted rehabilitation [21]. Liu et al. employed a combined approach integrating Virtual Reality Mirror Therapy (VRMT) with robot-assisted therapy for upper-limb rehabilitation [22]. Wang et al. developed a method that automatically generates posture trajectories for the affected limb by measuring limb orientations via IMUs, based on the concept of mirror therapy [23]. Therefore, enabling semi-autonomous, ADL task-guided rehabilitation training with robotic assistance is considered highly beneficial. Upper-limb exoskeletons can facilitate the movement of the affected limb, allowing daily ADLs to be transformed into coordinated bilateral training tasks aimed at restoring basic motor functions and establishing a solid foundation for recovery.

**Figure 1:**
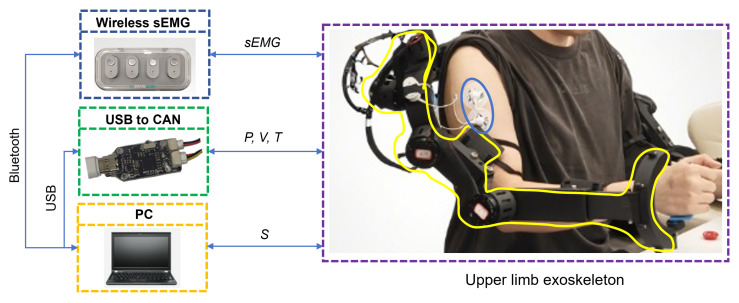
Schematic diagram of protocol for upper limb’s exoskeleton.

This paper proposes a task-guided bilateral exoskeleton mirror rehabilitation framework based on a cross-attention mechanism [24], which establishes an attention mechanism between sources of data, aiming to deliver precise and personalized interventions for early-stage stroke rehabilitation through multimodal biofeedback. Figure 1 shows the schematic diagram of the protocol for the upper limb’s exoskeleton. The proposed framework establishes a “healthy-side guidance–affected-side adaptation” bidirectional synergy mechanism:

- The unaffected limb’s exoskeleton captures kinematic features in real time.
- These kinematic features are fused with the temporal activation patterns of sEMG signals from the affected limb using a cross-attention network.
- The network dynamically generates synergistic control parameters for mirror rehabilitation tasks.
- The DMP algorithm decouples the spatiotemporal features of the healthy limb’s trajectory to generate an adaptable rehabilitation trajectory for the affected limb.
- A compliant control strategy under dynamic constraints is designed to accommodate individual differences in motor dysfunction, enabling dynamic compensation for abnormal postures while ensuring that joint torques remain within safe limits.

## 2. Materials and Methods

A mirror plane is defined as the sagittal plane P:x=0 (the y-z plane). The mapping from the unaffected side motion $y_{h}\left(t\right)$ to the affected side is given by the following equation:

(1) $$y_{p}^{\mathrm{mirror}}\left(t\right)=\left[\begin{array}{ccc} -1 & 0 & 0\\ 0 & 1 & 0\\ 0 & 0 & 1 \end{array}\right]y_{h}\left(t\right)$$

### 2.1. DMPs

DMPs are a representative method for simulating robot motion trajectories through imitation learning [25,26]. For robotic mirror therapy, the imitation characteristics of DMPs precisely meet the requirement for the robot to imitate the healthy limb (HL) behavior. Furthermore, DMPs have been widely used in human–robot coupling systems. Therefore, a combined scheme based on DMP-based trajectory generation is expected to improve therapeutic efficacy.

An adaptive DMP-based controller was developed to facilitate assisted motion of the exoskeleton, enabling accurate prediction and smooth synchronization of the robot’s motion trajectory. This achieves imitation of the master arm’s motion patterns by the follower arm, aligning with the requirements of mirror therapy. Although these methods achieve satisfactory performance in assisting human motion to complete prescribed tasks, they cannot accommodate patients’ abnormal postures or personalized needs.

Simple motion trajectories can be modeled using DMPs. Robots can learn manipulation skills from human demonstrations and extend human-like skills. In this way, human demonstrations can naturally and intuitively convey desired trajectory curves to the robot. Similar to trajectory planning, directly replicating the HL’s stiffness to the robot for Impaired Limb (IL) rehabilitation is not beneficial [27]. Ideally, both the robot’s trajectory and stiffness could be simultaneously optimized in this scheme. However, current research efforts are limited to demonstrating its feasibility in robotic mirror therapy [28].

### 2.2. Dynamic Mirror Ratio Controller

Traditional mirror therapy suffers from the limitations of a static coupling mechanism during movement mapping, primarily characterized by fixed mapping intensity and an inability to adaptively adjust based on the patient’s real-time motor capacity. To overcome this technical bottleneck, this paper innovatively proposes a dynamic mirror ratio control method. This method leverages a cross-attention network to compute a time-varying mirror ratio coefficient λ(t)∈[0,1] in real time and deeply couples it into the exoskeleton’s impedance control loop. This coefficient dynamically quantifies the intensity of the mapping from the motion of the unaffected limb to the training of the affected limb.

(2) $$K_{d}\left(t\right)=K_{base}\cdot \left(1-\lambda (t)\right)$$

(3) $$B_{d}\left(t\right)=B_{base}\cdot \left(1+\alpha \lambda (t)\right)\left(\alpha =0.5\right)$$

The above formula indicates that, when λ increases,

- $K_{d}$ (stiffness) decreases, requiring the patient to actively exert force to overcome positional deviations, thereby promoting neuroplasticity.
- Simultaneously, $B_{d}$ (damping) increases, suppressing the exoskeleton’s motion speed to prevent tremors or uncontrolled acceleration caused by insufficient muscle strength.

#### 2.2.1. Dual-Stream Encoding Architecture

To capture the specificity of heterogeneous data modalities, we employ a dual-stream encoding architecture. Human kinematic features (e.g., position, velocity) exhibit structured, low-dimensional, and high-signal-to-noise-ratio characteristics. For instance, joint angle sequences directly reflect the geometric trajectories of limb movement, with smooth variations adhering to mechanical dynamics. In contrast, sEMG signals manifest as non-stationary high-dimensional bioelectric signals prone to interference [29,30]. Key challenges include burstiness (e.g., sharp signal spikes at movement initiation) and signal attenuation due to muscle fatigue [31]. Directly mixing inputs into a single encoder would cause the following:

- Misalignment between continuous kinematics and impulsive sEMG features in latent space;
- High-frequency sEMG noise contaminating kinematic representations, degrading the trajectory and predictions [32,33];
- Redundant parameter allocation across modalities, increasing training complexity.

Specifically, we process the user’s unaffected limb kinematic data and affected limb physiological signals separately to avoid interference between heterogeneous data modalities. These are termed the kinematic encoding stream (KineEncoder) and the physiological signal encoding stream (BioEncoder).

The kinematic data of the unaffected limb mainly includes joint angles $q_{H}\left(t\right)\in R^{5}$ and joint velocities ${\dot{q}}_{H}\left(t\right)\in R^{5}$. These are normalized to [−1,1] respectively, and then concatenated into $X_{h}\left(t\right)\in R^{10}$. Since the self-attention mechanism of transformers [34] inherently lacks awareness of sequence order, positional encoding is used to inject position information into the sequence data. A four-layer transformer is adopted for the temporal modeling of motion information.

For an input sequence X∈$R^{T\times d}$, the output of the *l*-th layer is calculated as follows:

(4) $$Q^{l}=X^{\left(l-1\right)}W_{Q}^{l},\;K^{l}=X^{\left(l-1\right)}W_{K}^{l},\;V^{l}=X^{\left(l-1\right)}W_{V}^{l}$$

(5) $${\mathrm{Attn}}^{l}=\mathrm{Softmax}\left(\frac{Q^{l}{\left(K^{l}\right)}^{⊤}}{\sqrt{d}}\right)V^{l}$$

(6) $$X_{\mathrm{mid}}^{l}=\mathrm{LayerNorm}\left(X^{\left(l-1\right)}+{\mathrm{Attn}}^{l}\right)$$

(7) $$X^{l}=\mathrm{LayerNorm}\left(X_{\mathrm{mid}}^{l}+\mathrm{FFN}\left(X_{\mathrm{mid}}^{l}\right)\right)$$

The physiological signals primarily consist of eight-channel sEMG signals sEMG(t), which are generated during muscle contraction. Their time domain characteristics reflect muscle activation states and fatigue features. A bandpass filter is applied to remove noise from the raw signals. Subsequently, full-wave rectification and low-pass filtering are used to extract the signal envelope. Finally, normalization is performed:

(8) $$s_{\mathrm{norm}}\left(t\right)=\frac{s\left(t\right)-\mu_{\mathrm{baseline}}}{\sigma_{\mathrm{baseline}}}$$

where $\mu_{\mathrm{baseline}}$ and $\sigma_{\mathrm{baseline}}$ denote the mean and standard deviation of baseline resting-state signals.

In a biosignal-encoding pipeline that uses the local receptive fields and weight-sharing properties of the Convolutional Neural Network (CNN) [35], two convolutional layers extract transient amplitude variations and root mean square (RMS) changes to identify the onset of muscle activation, fatigue levels, and other physiological states. Given the significant temporal dependencies in muscle activity (for example, contraction–relaxation cycles and cumulative fatigue effects), a bidirectional Gated Recurrent Unit (GRU) is adopted for temporal modeling [36].

The forward GRU computation at timestep *t* is as follows:

(9) $$z_{t}=\sigma \left(W_{z}\cdot \left[h_{t-1},x_{t}\right]\right)$$

(10) $$r_{t}=\sigma \left(W_{r}\cdot \left[h_{t-1},x_{t}\right]\right)$$

(11) $${\tilde{h}}_{t}=\tanh\left(W\cdot \left[r_{t}⊙h_{t-1},x_{t}\right]\right)$$

(12) $$h_{t}=\left(1-z_{t}\right)⊙h_{t-1}+z_{t}⊙{\tilde{h}}_{t}$$

The backward GRU processes the sequence in reverse order. The final output is the concatenation of bidirectional states:

(13) $$H_{\mathrm{bi}}=\left[\vec{H}⊕\overset{\leftarrow }{H}\right]$$

#### 2.2.2. Cross-Modal Attention Fusion Mechanism

Building upon the dual-stream encoding architecture, we introduce a cross-modal attention mechanism to capture latent relationships between kinematic and physiological signals. This mechanism enables weighted fusion across modalities, achieving feature-level complementarity and enhancement [37,38]. It accurately reflects the real-time state of the affected limb, supporting dynamic mapping adjustments.

The kinematic features $H_{h}\in R^{T\times 256}$ from the kinematic encoder, and the sEMG features $H_{p}\in R^{T\times 128}$ from the biological encoder. The cross-attention module generates fused features that align the user’s movement intent with safe exoskeleton operation (avoiding hazardous joint angles/velocities):

(14) $$H_{\mathrm{fusion}}=\mathrm{CrossAttn}\left(E_{h}\left(q_{h}\right),E_{p}\left({\mathrm{sEMG}}_{p}\right)\right)$$

The dynamic coupling coefficient is then computed via a fully connected layer:

(15) $$\lambda \left(t\right)=\sigma \left(W_{\lambda }\cdot \mathrm{MLP}\left(H_{\mathrm{fusion}}\right)\right)$$

### 2.3. Control System and Algorithm

The dynamics of the exoskeleton mechanism follow the equation

(16) $$M_{d}\left(\ddot{q}-{\ddot{q}}_{d}\right)+D_{d}\left(\dot{q}-{\dot{q}}_{d}\right)+K_{d}\left(q-q_{d}\right)=\tau_{ext}$$

Parameters:

$M_{d},D_{d},K_{d}:$ The desired inertia, damping, and stiffness matrices;

$q_{d},{\dot{q}}_{d},{\ddot{q}}_{d}:$ The joint angle, velocity, and acceleration generated by DMPs;

$\tau_{ext}:$ The interaction torque between the user and environment.

For upper-limb rehabilitation tasks, the target trajectory is defined as a smooth motion curve in joint space. A rehabilitation action comprises *N* key points, with the end-effector trajectory expressed as

(17) $$T=\{P_{t}\in R^{3}|t=1,\ldots ,T\}$$

(18) $$P_{t}={\left[x_{t},y_{t},z_{t}\right]}^{T}$$

$P_{t}$: The position of the end effector at time *t*; $x_{t},y_{t},z_{t}$: 3D Cartesian coordinates of the end effector.

#### 2.3.1. Trajectory Planning

DMPs can generate adjustable trajectories for one side of the exoskeleton based on the trajectory of the other side, allowing personalized trajectory generation to adapt to individual differences. DMPs can also learn the dynamic characteristics of an action (including velocity and acceleration in addition to position). During exoskeleton mirror rehabilitation, trajectory planning is categorized into two types.

- Bimanual trajectory planning based on human symmetry plane: This transfers the unaffected limb’s motion to the affected limb through mirroring. The exoskeleton drives the affected limb to perform symmetric movements relative to the unaffected limb.
- Bimanual asymmetric task trajectory planning: This engages the affected limb’s motor participation for better generalization to daily activities.

In early rehabilitation stages, the affected limb has limited mobility [39] and requires guidance from the unaffected limb [40]. The unaffected limb drives its exoskeleton to generate appropriate trajectories for the affected exoskeleton, preventing potential harm. DMPs generate trajectories for asymmetric tasks, focusing on discrete movements for robotic trajectory planning.

(19) $$\tau^{2}\ddot{y}=\alpha_{y}\left(\beta_{y}\left(g-y\right)-\tau \dot{y}\right)+f$$

τ: Time constant;

$\alpha_{y}$, $\beta_{y}$: Constants;

*g*: Target position;

*f*: Nonlinear shaping function.

The nonlinear function is shown as

(20) $$f\left(t\right)=\frac{\sum_{i=1}^{N}\Psi_{i}\left(t\right)w_{i}}{\sum_{i=1}^{N}\Psi_{i}\left(t\right)}$$

$\Psi_{i}:\mathrm{Basis}\mathrm{functions}$;

$w_{i}:\mathrm{Corresponding}\mathrm{weights}$;

N:Numberofbasisfunctions.

Discrete DMP formulation:

(21) $$f\left(x,g\right)=\frac{\sum_{i=1}^{N}\Psi_{i}\left(x\right)w_{i}}{\sum_{i=1}^{N}\Psi_{i}\left(x\right)}x\left(g-y_{0}\right)$$

Constraint:

(22) $$\tau \dot{x}=-\alpha_{x}x$$

$\alpha_{x}:\mathrm{Constant}$.

Gaussian basis functions:

(23) $$\Psi_{i}\left(x\right)=\exp\left(-h_{i}{\left(x-c_{i}\right)}^{2}\right)=\exp\left(-\frac{1}{2\sigma_{i}^{2}}{\left(x-c_{i}\right)}^{2}\right)$$

$\sigma_{i}:\mathrm{Gaussian}\mathrm{width}$;

$c_{i}:\mathrm{Center}\mathrm{point}$.

The exoskeleton trajectory generation principles are as follows:

1. Minimize the position tracking error between the ideal and actual trajectories;
2. Ensure smooth joint motor angles, velocities, and accelerations to prevent secondary injury;
3. Adapt to individual anatomical differences and varying rehabilitation-stage mobility ranges.

#### 2.3.2. Algorithm

**Figure 2:**
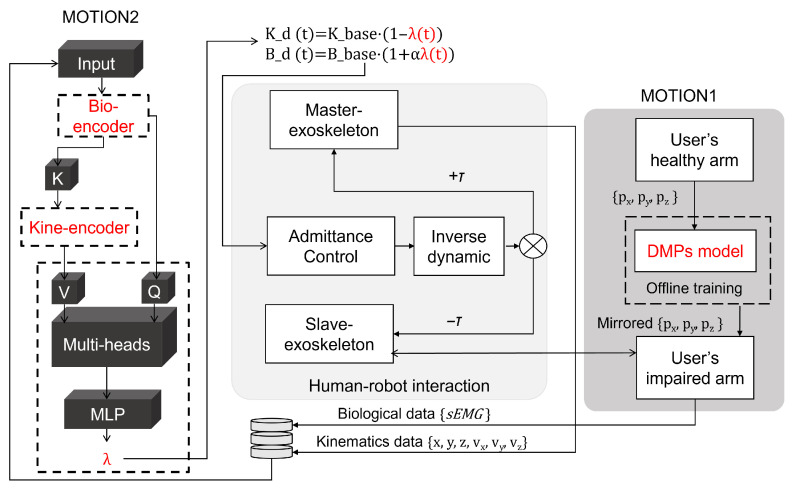
Control block diagram of the system.

The motion trajectory of the unaffected upper limb is processed by a transformer encoder to extract multi-joint synergistic movement features (see Figure 2). This model employs a four-layer self-attention mechanism to capture long-term temporal dependencies. The physiological signals from the affected limb are processed by a bidirectional GRU encoder to extract forward–backward temporal features. The cross-attention mechanism achieves dynamic mapping through the following steps.

For the kinematic encoder, the input consists of kinematic features from the unaffected side, including the positions and velocities of five joint motors: $X_{h}\left(t\right)=\left[\begin{array}{c} q_{H1},q_{H2},\ldots ,q_{H5},{\dot{q}}_{H1},{\dot{q}}_{H2},\ldots ,{\dot{q}}_{H5} \end{array}\right]\in R^{10}$. A four-layer transformer encoder with four-head attention is adopted, with a hidden-layer dimension: d = 64. The final output is a temporal contextual feature $H_{h}\in R^{T\times d}$. For the affected-side sEMG signal encoder, the input is the physiological signal sequence: $\mathrm{sEMG}\left(t\right)=\left[v_{1}\left(t\right),v_{2}\left(t\right),\ldots ,v_{8}\left(t\right)\right]\in R^{8}.$ A bidirectional GRU architecture is utilized, with a hidden layer dimension of d/2 (32 dimensions per direction). This is followed by layer normalization and a dropout layer (*p* = 0.2). The output is a temporal feature $H_{p}\in R^{T\times d}$.

#### 2.3.3. Offline Training

**Figure 3:**
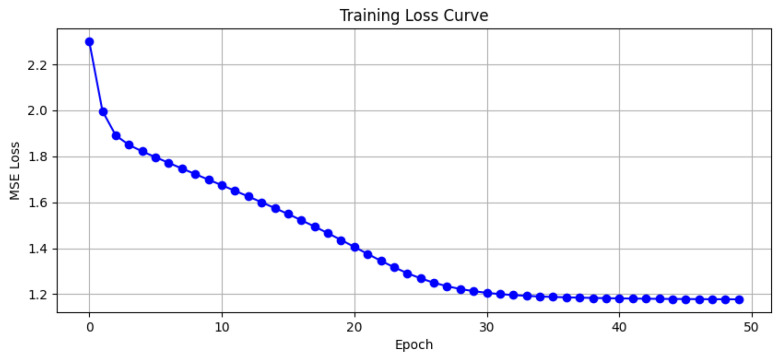
The training curve in 50 epochs.

Using input trajectory data and predefined DMP parameters, the first output is generated: the DMP imitation learning trajectory for the unaffected side’s exoskeleton. This trajectory undergoes a mirror coordinate transformation in the human sagittal plane (x = 0) to obtain the ideal motion trajectory for the exoskeleton of the affected side. This constitutes the second output. The joint angles and velocities of the exoskeleton on the unaffected side are input into the kinematic encoder, revealing kinematic features $H_{h}\in R^{T\times 256}$. The sEMG data from the affected side are input into the biological encoder, yielding biological features $H_{p}\in R^{T^{\prime }\times 128}$. These two features are fed into the cross-attention module to compute the dynamic mirroring ratio coefficient λ(t), and Figure 3 illustrates the model’s loss decrease during training.

#### 2.3.4. Admittance Control

To enable the exoskeleton to comply with human guidance while providing stable, predictable interaction behavior, an inverse dynamics framework with feedforward compensation is adopted:

(24) $$\tau =M\left(q\right){\ddot{q}}_{r}+C\left(q,\dot{q}\right){\dot{q}}_{r}+G\left(q\right)+J^{T}F_{\mathrm{ext}}$$

τ: The joint torque vector;

M(q): The inertia matrix;

$C(q,\dot{q})$: The Coriolis and centrifugal force matrix;

G(q): The gravity vector;

*J*: The Jacobian matrix;

$F_{ext}$: The external force vector.

The reference acceleration is computed as

(25) $${\ddot{q}}_{r}={\ddot{q}}_{d}-M_{d}^{-1}\left[D_{d}\left(\dot{q}-{\dot{q}}_{d}\right)+K_{d}\left(q-q_{d}\right)\right]$$

${\ddot{q}}_{d},{\dot{q}}_{d},q_{d}$: The desired acceleration, velocity, and position;

$M_{d}$, $D_{d}$, $K_{d}$: The desired inertia, damping, and stiffness matrices.

The task-space admittance control law is defined by

(26) $$M_{d}\left({\ddot{x}}_{d}-\ddot{x}\right)+B_{d}\left({\dot{x}}_{d}-\dot{x}\right)+K_{d}\left(x_{d}-x\right)=F_{\mathrm{ext}}$$

$x_{d},{\dot{x}}_{d},{\ddot{x}}_{d}:$ The desired end-effector position, velocity, and acceleration;

$x,\dot{x},\ddot{x}:$ The actual end-effector position, velocity, and acceleration;

$B_{d}:$ The task-space damping matrix.

### 2.4. Experiment

#### 2.4.1. Experiment Setup

Motion 1: Passive assist mode. During the initial rehabilitation stage, the affected limb exhibits minimal voluntary mobility. Consequently, movement is initiated by the unaffected limb that drives the contralateral exoskeleton. Upon reaching the target position, the contralateral exoskeleton applies impedance forces to the unaffected limb. To ensure minimal trajectory tracking error during this guidance, the contralateral exoskeleton operates in a high-stiffness mode while simultaneously collecting kinematic data. Currently, the affected side’s exoskeleton generates an imitation trajectory via DMPs, driving the affected limb toward the target position.

Motion 2: Active-adaptive cooperative mode. As the patient regains partial muscle strength in the affected limb, allowing the initiation of basic movements, but showing coordination deficits, the patient can actively trigger motion using residual sEMG signals from the affected limb. However, the exoskeleton must provide dynamic assistance to overcome insufficient force generation and motor discoordination. When the intensity of the sEMG signal from the affected limb muscles exceeds three times the baseline level of rest, the system detects motor intent and initiates the bilateral cooperative mode.

For the contralateral exoskeleton, the mode progressively transitions from a high-stiffness to a medium–low-stiffness mode. It temporarily increases stiffness only after detecting significant lag in the movement of the affected limb, which is defined as a velocity difference that exceeds 25% to suppress the excessive compensatory motion of the unaffected limb. The ipsilateral exoskeleton (affected side) implements an EMG–mechanics hybrid control strategy. The intensity of the patient’s EMG signals is mapped in real time to the magnitude of the assistive torque provided by the exoskeleton. A stronger EMG signal results in proportionally less active exoskeleton assistance, thus encouraging the patient to progressively exert more voluntary force.

For the starting posture, both arms are fitted with the upper-limb exoskeleton and positioned at the designated start location on the table, as set by the assistant. The unaffected limb autonomously executes the movement from the start point to the target point at a controlled velocity (0.1–0.3 m/s). For the affected limb, a dynamic mirror ratio controller generates a coefficient λ to modulate the exoskeleton’s impedance parameters. During the arm movement towards points a, b, and c, sEMG signals from both limbs are acquired under the complete movement pattern. The RMS value of the acquired sEMG signals is calculated. The variation in λ is utilized to establish a control group for analysis.

#### 2.4.2. Hardware

Table 1 summarizes the hardware setup, including the joint modules, degrees of freedom, peak torque, and adjustment mechanisms. For each side of the exoskeleton, the driving force is provided by five direct-drive motors, including one DM-J8009 (DaMiao, Shenzhen, China) and four DM-J4340 (DaMiao, China). The exoskeleton accommodates anthropometric variations through two key adaptations: Shoulder width adjustment is achieved via a linear slider mechanism (53 to 193 mm range for each side), while the forearm segment length adaptation utilizes telescopic sleeves (260 to 300 mm) secured by passive locking pins at 10 mm intervals. For this experimental setup, wrist joint rotation remains disabled, with the end-effector position defined as the centroid of the distal plane of the wrist joint complex. The sEMG acquisition device used is the BC124 (BrainClos, Shenzhen, China).

#### 2.4.3. Multimodal Dataset

Five healthy participants were recruited, aged 20–25 years, with a mean age of 23 ± 1.6 years, including four males and one female, without daily upper-limb pain or activity limitations. Upper-limb joint angles were recorded via joint motor encoders. sEMG signals were acquired using an 8-channel system sampled at 2000 Hz. The total task duration *T* was measured from movement initiation until stable positioning within the target region was achieved and maintained. This metric quantifies the spatiotemporal coordination of the end-effector trajectories of both limbs in 3-dimensional space. A higher covariance value indicates greater synchrony between the trajectories of the unaffected and affected limbs. A value approaching 1 signifies highly coordinated bilateral movement, suggesting a successful promotion of symmetry through the rehabilitation strategy. A value approaching 0 indicates a lack of association between the limb movements, potentially signifying compensatory movements or control failure.

The temporal axes of the bilateral trajectories are first aligned using dynamic time warping. Subsequently, the covariance is calculated for each dimension x,y,z of the unaffected limb trajectory coordinates relative to the affected limb trajectory coordinates.

## 3. Results

**Figure 4:**
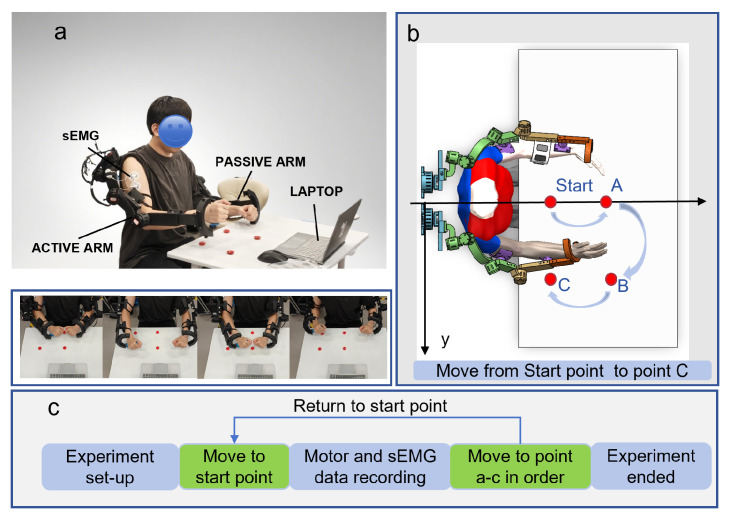
Panel (**a**) illustrates the structural configuration of the bilateral upper-limb exoskeleton system. Panel (**b**) displays the motion trajectory of the arm-driven exoskeleton from a top–down perspective. Panel (**c**) schematically outlines the experimental workflow.

As shown in Figure 4, the system configuration, motion trajectory, and experimental workflow are illustrated.

**Figure 5:**
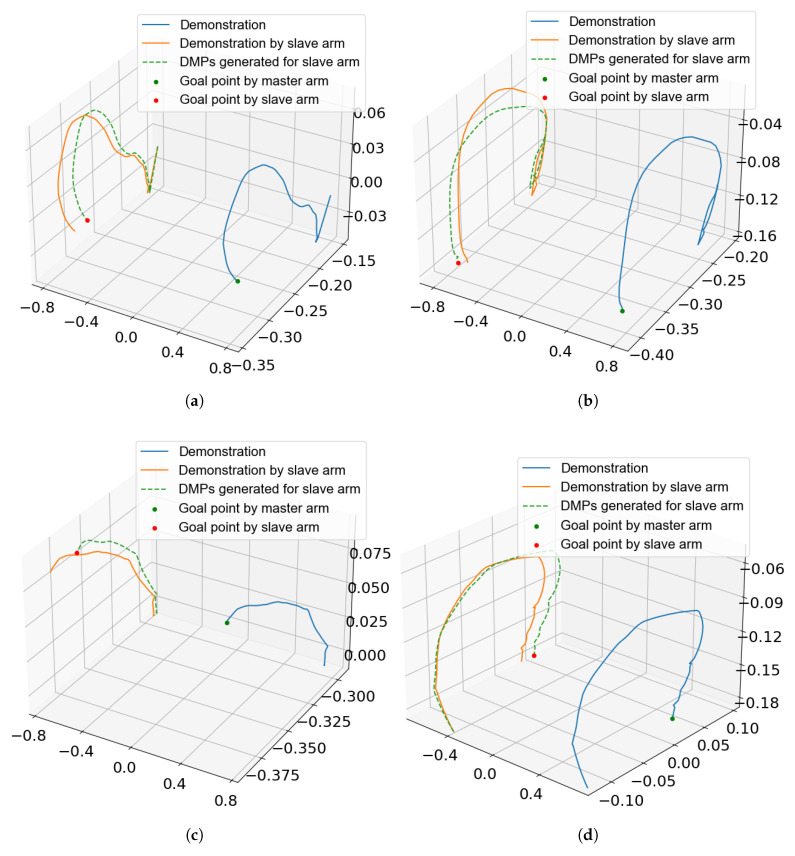
The figure illustrates the raw trajectory of the exoskeleton’s active endpoint, the mirrored trajectory under ideal conditions, and the actual trajectory generated via DMPs. (**a**) Endpoint trajectory from the start position to point A; (**b**) trajectory between points A and B; (**c**) trajectory between point B and C; (**d**) trajectory between point C and Start.

In Figure 5, a single-subject case is presented, where panels (a)–(d) demonstrate the motion trajectories of subject 1 from the start position to predefined targets A, B, and C, respectively.

## 4. Discussion

**Figure 6:**
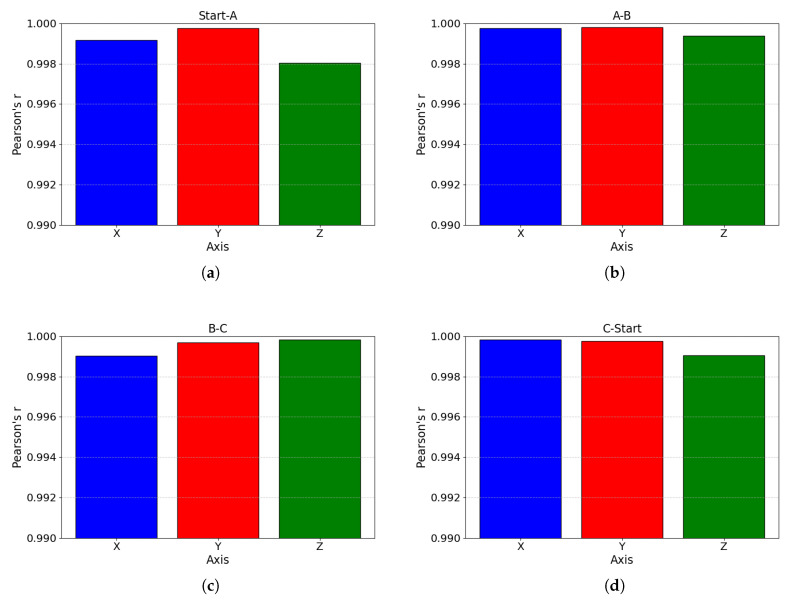
The blue, red, and green colors represent the covariance magnitudes (expressed as Pearson correlation coefficients) along the *x*-, *y*-, and *z*-axes of the bilateral exoskeleton endpoints, respectively. Panel (**a**) displays the values during movement from the start point to point A; panel (**b**) displays the values in the trajectory from point A to point B; panel (**c**) displays the values in the trajectory from point B to point C; and panel (**d**) displays the values in the trajectory from point C to point Start.

**Figure 7:**
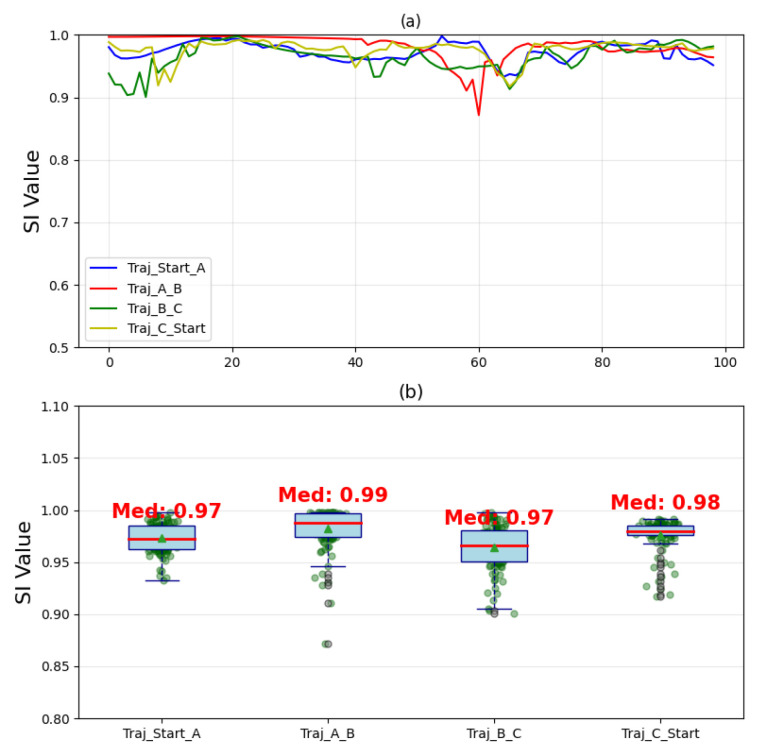
The variation trend of mirror coefficient values and their distribution characteristics. (**a**) illustrates the variation of the SI values for each of the four trajectories across the temporal sampling points; (**b**) presents the distribution characteristics of the SI values for each trajectory.

In Figure 6, the bilateral trajectory covariance (expressed as Pearson correlation coefficients) for all four trajectories (Start-A, A–B, B–C, and C-Start) exceeds 0.998 across all three spatial dimensions. In Figure 7, the analysis reveals the following:

- Trajectory Start–A: Symmetry index (SI) median = 0.97 (range: 0.93–1.00), with the data concentrated between 0.95 and 1.00.
- Trajectory A–B: SI median = 0.99 (range: 0.87–1.00), showing dispersed distribution and outliers, indicating mild mirroring asymmetry. However, the SI values remain close to 0.90, suggesting minimal trajectory fluctuations.
- Trajectory B–C: SI median = 0.97 (range: 0.88–1.00), with distribution patterns similar to those of trajectory A–B but with greater dispersion.
- Trajectory C–Start: SI median = 0.98 (range: 0.92–1.00).

**Figure 8:**
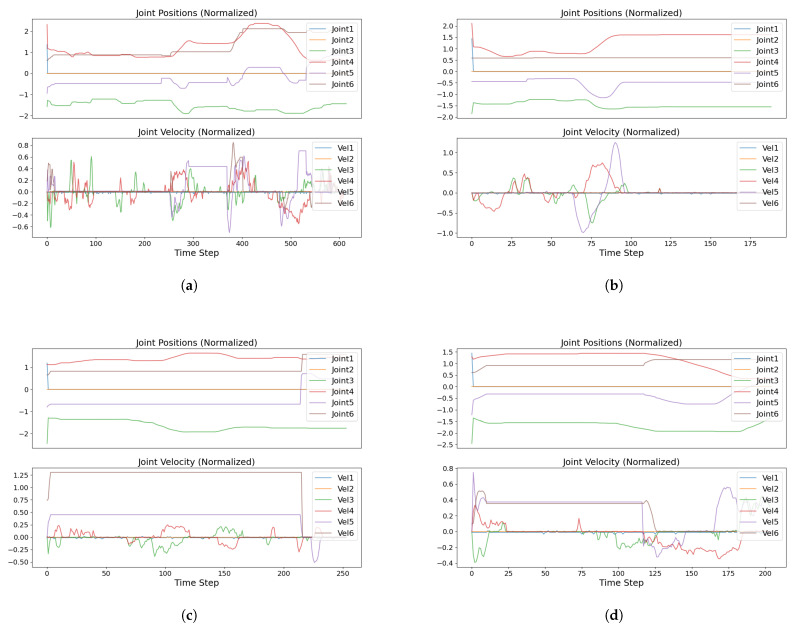
Panel (**a**) presents the velocity and position of the end effector during movement from the start position to target A. Panels (**b**–**d**), respectively, demonstrate the RMS trajectories for the A–B movement, the B–C movement, and the return path from C to the start position.

Figure 8 presents the velocity and position of the end effector during the movement phases, along with RMS trajectories for the A–B, B–C, and return movements.

**Figure 9:**
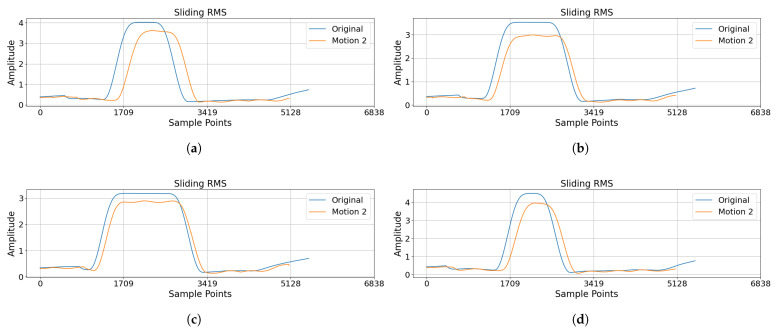
Panel (**a**) presents the EMG RMS variation profile during movement from the start position to target A. Panels (**b**–**d**), respectively, demonstrate the RMS trajectories for the A–B movement, the B–C movement, and the return path from C to the start position.

The experimental results demonstrate that, in Figure 9, the highest RMS values of the compared trajectories exhibit reduction ratios of 10.8%, 16.1%, 9.8%, and 12.4% in four movement segments, respectively. The mean RMS reduction ratio for all the segments is 12.3%. Compared to the original λ values, the RMS of the sEMG amplitudes was consistently lower when calculated using the dynamic mirror ratio controller in Motion2. Experiments with healthy subjects demonstrated that dynamic λ control enabled bilateral trajectory co-variation. In accordance with stroke rehabilitation theory, the motion synchronization achieved by the exoskeleton may provide neural activation conditions for patients. The primary goal of this stage of the research was to validate the functionality of the core algorithm under ideal conditions (i.e., healthy subjects), rather than to evaluate clinical efficacy. The current results merely indicate the system’s technical potential to support rehabilitation and do not address clinical application.

## 5. Conclusions

A cross-attention mechanism is introduced into mirror therapy to achieve real-time alignment between patient motor capability and the required assistance level. Through the online adjustment of impedance parameters, the proposed approach addresses a key limitation of traditional mirror therapy, which typically focuses on positional tracking while neglecting force-related interactions.

This study proposes a novel dynamic mirror therapy framework for bilateral upper-limb exoskeleton rehabilitation, addressing the limitations of traditional static mirroring approaches. By integrating a cross-attention network and adaptive DMPs, the system enables the real-time modulation of mirroring intensity. The primary contributions include the following.

Patient-specific motion mapping via cross-attention: The fusion of the kinematic features of the unaffected limb and surface electromyography signals of the affected limb allows the dynamic calculation of a mirroring ratio coefficient λ(t)∈[0,1] using a mechanism of cross-attention, thereby achieving patient-specific motion mapping. The generation of adaptable trajectories for the affected limb is achieved using DMPs, which preserve spatiotemporal coordination while accommodating asymmetric motor capabilities. The adjustment of exoskeleton impedance parameters is based on multimodal feature extraction. Compared to fixed-impedance methods, this adaptive approach results in a reduction in the RMS of EMG signals. While this framework demonstrates potential for early-stage stroke rehabilitation, current limitations include limited adaptability to severe spasticity and unverified long-term neuroplastic effects. Our experiments also revealed that, when the exoskeleton end effector moves to different preset target points, asymmetry persists in the trajectory curves generated for the contralateral side. The potential reasons for this include deviations induced during exoskeleton donning and alignment, as well as suboptimal parameter settings within the DMPs used to generate the contralateral trajectory curves.

Future work will focus on optimizing the mechanical structure of the exoskeleton, particularly by increasing the DOF of the shoulder joint, integrating vision modules, increasing the number of electromyography (EMG) sensors, and incorporating electroencephalography (EEG)-based cortical activity monitoring to establish a comprehensive multimodal fusion dataset. Additionally, clinical validation will be extended to patients with varying degrees of upper-limb dysfunction.

## Figures and Tables

**Table 1:** The joint module configuration and technical details for a single-arm assembly of one side of this upper limb’s exoskeleton.

Joint Module	DOF Type	Peak Torque	Adjustment Mechanism
Shoulder girdle	1 Active DOF	40 Nm	/
Shoulder complex	3 Active DOFs	27 Nm	Linear slider
Elbow joint	1 Active DOF	27 Nm	Telescopic sleeve
Wrist joint complex	2 Passive DOFs	/	Telescopic sleeve

## Data Availability

The data is unavailable due to privacy and ethical restrictions.

## Abbreviations

The following abbreviations are used in this manuscript:

DMPs	Dynamic Movement Primitives
sEMG	Surface Electromyography
DOF	Degree of Freedom
CNN	Convolutional Neural Network
GRU	Gated Recurrent Unit
RMS	Root Mean Square
